# Industrial and Personal Hygiene

**Published:** 1908-09

**Authors:** 


### BUFFALO MEDICAL JOURNAL.

A Monthly Review of Medicine and Surgery.

*EDITOR:*

WILLIAM WARREN POTTER, M. D.

All communications, whether of a literary or business nature, books for review and exchanges, should be addressed to the editor 238 DELAWARE AVE., BUFFALO, N. Y

VOL. LXIV. SEPTEMBER, 1908. No. 2

### Industrial and Personal Hygiene.

PRESIDENT ROOSEVELT has demonstrated many times his concern for the welfare of the people, and especially his interest in the prevention of disease through the establishment of efficient sanitary measures. Perhaps one of the most conspicuous instances in which he has manifested this concern is in the establishment of what is known as "The President's Homes Commission," consisting of fifteen men and women,—to be exact, thirteen men and two women,—more or less noted for their philanthropic proclivities and scientific knowledge.

At the head of this commission stands the name of Brigadier General George M. Sternberg, Surgeon General United States Army, retired, whose work on the lines of preventive medicine is conspicuous. The secretary is Dr. George M. Kober, professor of hygiene at Georgetown University, also noted as a scientific man. Dr. Kober also is chairman of the committee on Social Betterment, a subcommittee of the commission, and his committee has issued recently a report on industrial and personal hygiene that possesses great interest and which becomes of importance in the study of problems connected with these topics. The report is a standard octavo brochure of 175 pages, printed on soft white paper, and is well indexed.

About 100 pages of the report are devoted to the consideration of industrial hygiene in which the morbidity and mortality of wage-earners are dealt with in considerable detail, and statistics, taken from the census reports and elsewhere, are offered in support of the statements made. It is shown that many persons are engaged in occupations for which they are not physically fitted, while others ruin their health by vice, dissipation, improper food and insanitary home environment.

Curiously enough, an example is cited for which neither industry nor the individuals are to blame—namely, the anemia of the agricultural classes in Porto Rico, which, a few years ago, was attributed to their occupation and starvation, but it has been discovered recently that it was caused by the hookworm disease. The dust inhaling occupations, as is well known, cause or contribute to irritating diseases of the respiratory tract. The bearing of elevator dust in causing diseases of the air passages was worked out many years ago by Dr. Thomas F. Rochester, of Buffalo. Since that time it has become known that many other dust particles, habitually inhaled, cause respiratory diseases. Workers in metallic and mineral dust industries are specially liable to diseases of the throat and lungs.

All these and many other dangers such as vegetable dust, the dust from textile industries, animal dust, the infective matter in dust from rag, paper, wool and hair industries and lead dust are pointed out and dealt with in an intelligent and comprehensive manner. Special measures for the prevention of disease among wage-earners and for their protection are considered, as well as the sanitation of workshops and quarters for employees, the latter involving cubic air space, ventilation, temperature, humidity, lighting, while the prevention of spitting and many miscellaneous sanitary provisions are dealt with.

As bearing on the further question of prevention the report considers what the federal government may do for the promotion of the welfare of its employees; what the employer may do; what the general public may do; and, finally, what the employee may do to contribute to his own welfare.

The second part of the report deals with personal hygiene, in which questions relating to house and home, baths, clothing and arrangement of dress, undergarments, footwear, the teeth and the care of the special organs are treated of. The report advises that deep breathing in the open air or near an open window, by taking ten or twelve deep inspirations morning and evening, should be practised, which is believed to secure the thorough ventilation of all the air vesicles. In an appendix the health of employees in the government printing office at Washington is considered by Dr. William J. Manning, chief of the sanitary division of that office. An illustration of the Emergency Hospital in the government printing office is given.

The report as a whole is of an excellent nature, has been prepared with great care, and cannot fail to exert a useful influence over employer, employee, and the general public. It is to be hoped that it will be distributed at least very extensively to those employers, who have large numbers of wage-earners in charge.

